# Wood‐Inspired Cement with High Strength and Multifunctionality

**DOI:** 10.1002/advs.202000096

**Published:** 2020-12-23

**Authors:** Faheng Wang, Yuanbo Du, Da Jiao, Jian Zhang, Yuan Zhang, Zengqian Liu, Zhefeng Zhang

**Affiliations:** ^1^ Shi‐Changxu Innovation Center for Advanced Materials Institute of Metal Research Chinese Academy of Sciences Shenyang 110016 China; ^2^ Nano Science and Technology Institute University of Science and Technology of China Suzhou 215123 China; ^3^ Jihua Laboratory Foshan 528200 China; ^4^ School of Transportation Science and Engineering Harbin Institute of Technology Harbin 150090 China; ^5^ School of Materials Science and Engineering University of Science and Technology of China Hefei 230026 China

**Keywords:** bioinspiration, cement, ice templating, multifunctionality, strength

## Abstract

Taking lessons from nature offers an increasing promise toward improved performance in man‐made materials. Here new cement materials with unidirectionally porous architectures are developed by replicating the designs of natural wood using a simplified ice‐templating technique in light of the retention of ice‐templated architectures by utilizing the self‐hardening nature of cement. The wood‐like cement exhibits higher strengths at equal densities than other porous cement‐based materials along with unique multifunctional properties, including effective thermal insulation at the transverse profile, controllable water permeability along the vertical direction, and the easy adjustment to be water repulsive by hydrophobic treatment. The strengths are quantitatively interpreted by discerning the effects of differing types of pores using an equivalent element approach. The simultaneous achievement of high strength and multifunctionality makes the wood‐like cement promising for applications as new building materials, and verifies the effectiveness of wood‐mimetic designs in creating new high‐performance materials. The simple fabrication procedure by omitting the freeze‐drying treatment can also promote a better efficiency of ice‐templating technique for the mass production in engineering and may be extended to other material systems.

## Graphical abstract

By learning from natural wood, new cement materials with unidirectionally porous architectures are developed using a simplified ice‐templating technique. The wood‐like cement exhibits markedly higher strengths at equal densities than conventional cement materials. This is accompanied by a simultaneous achievement of multifunctional properties, including effective thermal insulation, controllable water permeability, and easy adjustment between hydrophilic and hydrophobic characteristics.

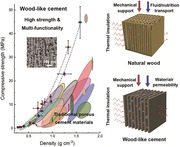

## 1 Introduction

Porous cement‐based materials possess a series of characteristic properties, e.g., low thermal conductivity for heat insulation, high sound absorbing efficiency along with outstanding permeability for air and water, and at the same time are lightweight and fire resistant.^[^ ^1^, ^2^, ^3^, ^4^ ^]^ These make them highly attractive and widespread in a range of applications, particularly where both mechanical and functional properties are required, like using as thermal insulation bricks, structural members, partitions, masonry infills, and pervious pavements. The porous cement‐based materials are most commonly fabricated following two different schemes, i.e., prefoaming by blending with preformed foam materials or mixed foaming by adding foaming agents during the forming process.^[^ ^4^, ^5^, ^6^, ^7^ ^]^ However, these methods invariably create randomly distributed pores in materials without any preferential orientation of structures or properties. Additionally, the pores are generally isometric in geometry and display rather coarse dimensions of some hundreds of micrometers to several millimeters.^[^ ^4^, ^5^, ^6^, ^7^ ^]^ Such an isotropic structural motif contrasts sharply to the property requirements in applications for porous cement‐based materials, which are often directional. For example, the thermal insulation materials for energy conservation, e.g., porous bricks used in external walls, need to bear compressive loads along the vertical direction, yet at the same time minimize thermal and sound conduction principally at the transverse profile.^[^ ^6^, ^7^, ^8^ ^]^ In comparison, the pervious pavements necessitate good load‐bearing ability and water permeability at the same configuration, i.e., along the vertical direction.^[^ ^9^ ^]^

However, it still remains a key challenge about how to achieve simultaneous enhancement of both mechanical and multifunctional properties, particularly along specific directions, in porous cement‐based materials. Additive manufacturing, as represented by 3D printing in form of direct ink writing, provides a viable approach for generating desired architectures with designed anisotropy in cement.^[^ ^10^, ^11^, ^12^, ^13^ ^]^ Nevertheless, its wide application, in practice, is strictly limited by the high processing cost, low production efficiency, and the difficulty in manipulating the rheological properties of cement inks for printing while maintaining a satisfied shape‐holding ability after printing. Additionally, the relatively weak interfaces between printed elements usually display a high damaging tendency,^[^ ^12^, ^13^ ^]^ thereby deteriorating the mechanical properties of 3D‐printed cement materials.

Taking lessons from nature offers increasing inspiration and opportunities for the development of new materials with improved performance.^[^ ^14^, ^15^, ^16^, ^17^ ^]^ As a naturally occurring porous material, the wood in plant stems features an outstanding combination of mechanical and multifunctional attributes, including robust mechanical support along with effective transport of water and nutrition along the vertical direction, and good thermal insulation properties at the transverse profile. These are largely reminiscent of the property requirements for porous cement‐based materials in applications. Such characteristics of natural wood have been revealed to stem essentially from its complex hierarchical architectures, which have been finely regulated at multiple length scales.^[^ ^18^, ^19^, ^20^ ^]^ In particular, the micropores involved in the wood cells, tracheids, vessels, and sieve tubes are principally orientated along the growth direction of plant, i.e., the vertical direction of wood stem.^[^ ^20^ ^]^ The unidirectionally porous architecture plays a key role in optimizing the anisotropic performance of wood, specifically by creating desired properties along required directions, including vertical stiffness, strength, and most abundant conduits for potent transport along with a good transversal insulation. It will be highly desirable for generating enhanced mechanical and multifunctional properties if the design principles of natural wood could be implemented in an effective way in exploiting new porous cement‐based materials.

## 2 Results

### 2.1 Formation of Wood‐Like Porous Cement

**Figure 1:**
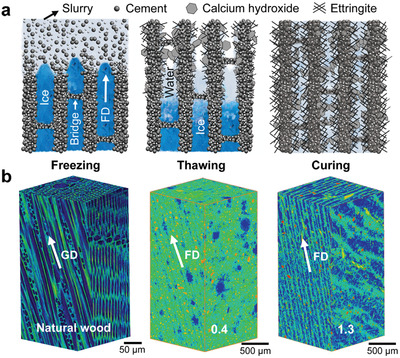
Formation and 3D architecture of wood‐like cement. a) Schematic illustrations about the formation micromechanisms of the wood‐like cement during the freezing, thawing, and curing processes. b) XRT volume renderings of the ice‐templated cement produced from cementitious slurries with W/C values of 0.4 and 1.3 with that of a *Betula schmidtii* birch wood as comparison. The pores in materials are indicated with blue color. FD and GD represent the freezing direction of ice and growth direction of wood, respectively.

**Figure** **1**a illustrates the formation process and micromechanisms of the wood‐like cement with unidirectionally porous architectures. Full details on the fabrication methods are given in the “Experimental Section.” The dual temperature gradients, i.e., from the bottom to the top in the mold and from the thinner end to the thicker end of the wedge, trigger the directional growth of ice crystals in the cementitious slurries into a series of parallel lamellae during the bidirectional freezing treatment,^[^ ^21^, ^22^ ^]^ as illustrated in Figure S1 (Supporting Information). In this process, the cement particles and additives are expelled from ice crystals into their spacing at the freezing front, and, by this, means are assembled into a long‐range‐ordered lamellar architecture. In particular, bridges may form between the cement lamellae because of the splitting of the dendritic tips of ice crystals, the engulfment of cement particles within the dendrites, and the subsequent healing of the tips.^[^ ^23^, ^24^ ^]^

The fully frozen bodies are then subject to a thawing process where the ice gradually melts at a temperature of 2 °C which is slightly higher than the freezing point of water. This allows for the in situ hardening of the cement by offering a source of water for its hydration reactions. As such, the ice‐templated architecture can be retained in the cement scaffold even after the removal of ice. The final curing treatment by immersing in water leads to the continuous hardening of the porous cement with immersing time by hydration reactions. An adequate hardening effect can be generated in the cement after curing for 28 days with further treatment for even longer time hardly leading to obvious additional hardening, as shown in Figure S2 (Supporting Information). Specifically, the hydration reactions produce a set of new minerals and gels in the cement, which have been identified principally as hexagon‐shaped calcium hydroxide, needle‐like ettringite, and calcium‐silicate‐hydrate gels.^[^ ^25^, ^26^, ^27^, ^28^, ^29^ ^]^ The formation mechanisms of these phases have been intensively explored with the detailed chemical reactions presented in the Supporting Information.^[^ ^27^ ^]^ In case of the current scaffold, theses phases primarily originate at the cement lamellae and grow into their spacing during the thawing and curing processes. The interpenetration and linkage between them play a role in enhancing the interconnection between lamellae, thereby promoting a better structural integrity of the porous cement.

X‐ray tomography (XRT) imaging clearly reveals the formation of unidirectional micropores between cement lamellae orientated in line with the freezing direction in the ice‐templated cement, as shown in Figure 1b. The content and thickness of these interlamellar pores display an increasing trend as the initial cementitious slurries get thinner with the water‐to‐cement ratio (W/C) varying from 0.4 to 1.3. The 3D structure of a *Betula schmidtii* birch wood is also presented in Figure 1b for comparison. It is seen that the ice‐templated cement resembles to a large extent the natural wood in terms of their unidirectionally porous architectures despite the large differences in the chemical compositions and characteristic dimensions of the pores. This represents some kind of implementation of the optimizing design principles of natural wood instead of a rigid replication of its structural characteristics. By contrast, the pores involved in the cement without ice‐templating treatment exhibit isometric geometries and a random distribution, as shown in Figure S3 (Supporting Information). In particular, there are large spherical voids with diameters exceeding 300 µm in the cement which may act as prime sites of damage to deteriorate the mechanical properties.^[^ ^30^, ^31^ ^]^ The comparison of wood‐like cement with the unfrozen one produced from the same slurries (with a constant W/C of 0.4) demonstrates a good effectiveness of the ice‐templating treatment in decreasing both the content and dimension of large voids together with the introduction of unidirectional pores. Large voids become even hardly discernible in the ice‐templated cement as W/C increases to 1.3.

### 2.2 Microstructural Characteristics

**Figure 2:**
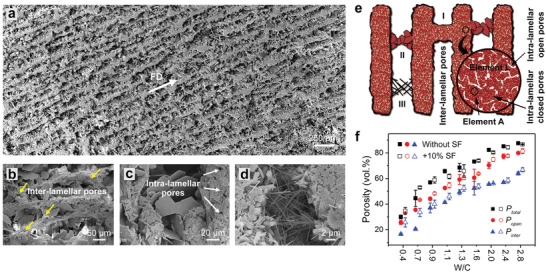
Microstructural characteristics of wood‐like cement. a) Cross‐sectional SEM images of the ice‐templated cement produced from slurries with a W/C of 1.3. b–d) SEM images of the interconnections between cement lamellae. b) Bridges and intersections formed during the freezing process, as indicated by the yellow arrows, along with the mineral products of hydration reactions of c) calcium hydroxide and d) ettringite. e) Schematic illustration about the different types of interconnections and pores in the ice‐templated cement. The circles indicate elements A and L for the formulation of strength using the equivalent element approach. f) Variations in the total porosity *P*_total_, open porosity *P*_open_, and interlamellar porosity *P*_inter_ in the cement with W/C in initial cementitious slurries. The data in panel (f) are obtained from at least three measurements for each set of samples and presented in form of mean ± standard deviation.

Scanning electron microscopy (SEM) imaging reveals that the unidirectional pores between lamellae in the ice‐templated cement are open and continuous, and encompass a large amount of interconnections bridging the lamellae at both sides of them, as shown in **Figure** **2**a–d. These interconnections can be classified into three primary types with markedly different characteristic dimensions: I) the bridges and intersections of lamellae having a width of 20–50 µm, which result from the engulfment of cement particles in the ice crystals during freezing (Figure 2b); II) the hexagon‐shaped calcium hydroxide with 30–70 µm in diameter and 1–3 µm in thickness (Figure 2c); and III) the needle‐like ettringite with 100–300 nm in diameter and several to over 10 µm in length (Figure 2d). The latter two minerals are the products of the hydration reactions of cement in the thawing and curing processes.^[^ ^27^ ^]^

In addition to the unidirectionally open pores, the cement lamellae per se are not fully dense but contain abundant pores with smaller dimensions which may be in either open or closed forms, as can be seen in Figure 2b–d. These pores have been demonstrated to form principally in the drying process of cement as a result of the dehydration of gels and the removal of water from originally water‐filling gaps between solid constituents.^[^ ^4^, ^27^, ^32^ ^]^ Therefore, the pores in the wood‐like cement can be classified into three types: interlamellar open pores, intralamellar open pores, and intralamellar closed pores, as illustrated in Figure 2e. Figure 2f presents the variations in the total porosity *P*_total_, open porosity *P*_open_, and interlamellar porosity *P*_inter_ in the ice‐templated cement made from cementitious slurries with different W/C. All these porosities display a monotonic rising trend as W/C increases with no clear differences discernible between the cement with and without silicon fume (SF) additions. Specifically, the total porosity can be regulated in a wide range from ≈30 to >80 vol% by controlling the solid load of slurries. This corresponds to a nominal density from 1.58 to 0.27 g cm^−3^ of the cement which can be more lightweight than water when W/C exceeds 0.7.

It is noted that the varying cooling rates with the distance from cold finger during the freezing process generally lead to differences in the thickness and spacing of lamellae in the ice‐templated scaffolds.^[^ ^23^, ^24^, ^33^, ^34^ ^]^ Nevertheless, such inhomogeneity has been revealed to mainly occur close to the bottom of mold, i.e., at the initial stage for the rapid growth of ice crystals, but become less evident as the solidification process reaches a relatively steady state.^[^ ^33^, ^34^ ^]^ For the current cement, the structural inhomogeneity is seen to concentrate to the end near the wedge, as shown in Figure S4 (Supporting Information), and is indiscernible over a range of several millimeters in the bulk (Figures 1b and 2a). Additionally, the interlamellar porosities have been measured for different sections of the cement at various positions along its height direction. As shown in Figure S5 (Supporting Information), the interlamellar porosities at almost all these sections locate well within the ranges for the entire cement with no obvious differences discernible between different positions for given W/C, clearly indicating a good structural homogeneity in the wood‐like cement. This results from the fact that the interlamellar porosity is principally governed by the content of water in initial slurries where water plays a similar role as a pore‐forming agent.

### 2.3 Mechanical Properties

**Figure 3:**
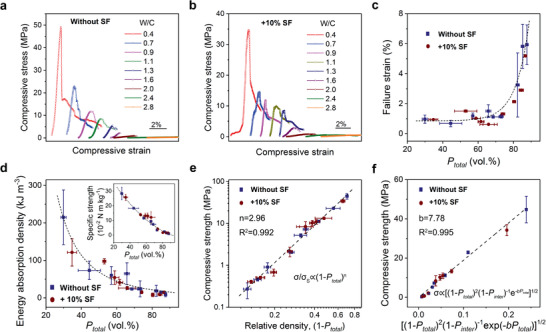
Mechanical properties of wood‐like cement. a,b) Representative compressive stress–strain curves of the wood‐like cement made from slurries with differing W/C a) without and b) with SF additions. c,d) Variations in the c) failure strain, d) energy absorption density, represented using the area under stress–strain curve until the peak stress, and specific strength (inset in panel (d)) as a function of the total porosity *P*_total_. The general varying trends are indicated by the dashed curves for clarity. e) Dependence of the compressive strength on the relative density in the wood‐like cement. f) Interpretation of the strength according to the equivalent element approach by taking different types of pores into account. The data in panels (c)–(f) are obtained from at least three measurements for each set of samples and presented in form of mean ± standard deviation.

**Figure** **3**a,b presents the representative compressive stress–strain curves of the wood‐like cement without and with 10 wt% SF additions produced from cementitious slurries with different W/C. The compressive strength is seen to decrease monotonically with rising W/C in the slurries, i.e., increasing porosity in the cement. The stress tends to exhibit obvious decrease after its peak value, implying the failure of cement in further bearing load. Indeed, the apparent nominal deformation of the wood‐like cement, particularly made from slurries with large W/C, after the peak stress is not true plasticity, but is caused by the continuous brittle collapse of its lamellae, as shown in Figure S6 (Supporting Information). As such, the mechanical properties, i.e., strength, failure strain, and energy absorption, are accessed on the stress–strain curves by examining up to the peak stresses. As shown in Figure 3c, the failure strain, i.e., the strain at the peak stress, demonstrates an increasing trend with the increase in total porosity. In contrast, the energy absorption density at failure, represented using the area under stress–strain curve until the peak stress, and the specific strength, i.e., strength normalized by nominal density, exhibit decreasing variations (Figure 3d). No obvious differences can be discerned between the mechanical properties of cement with and without SF additions when fixed porosities are concerned.

The strength *σ* of porous solids is principally determined by its porosity *P*. For an open‐celled foam, the strength can usually be correlated to its relative density with respect to a fully dense solid *ρ*_r_, i.e., the complementary of porosity with *ρ*_r_ = 1 − *P*. An empirically power‐law relationship exists as follows

(1) $$\begin{array}{c} \sigma /\sigma_{s}\propto {\left(1-P\right)}^{n} \end{array}$$

where *σ*_s_ denotes the strength of the fully dense solid. As shown in Figure 3e, the dependence of strength on the total porosity *P*_total_ in the current cement can be well captured by the above equation with the parameter *n* fitted to be 2.96. However, it fails to discern the effects of the three types of pores in the cement in view of their distinctly different characteristics. In particular, the mechanical role of its unidirectionally porous architectures cannot be clearly interpreted. Based on the linear elastic fracture mechanics, the strength of the wood‐like cement can be formulated by taking all these different pores into account using an equivalent element approach (Figure 3f), as is discussed in the following sections.

### 2.4 Multifunctional Properties

**Figure 4:**
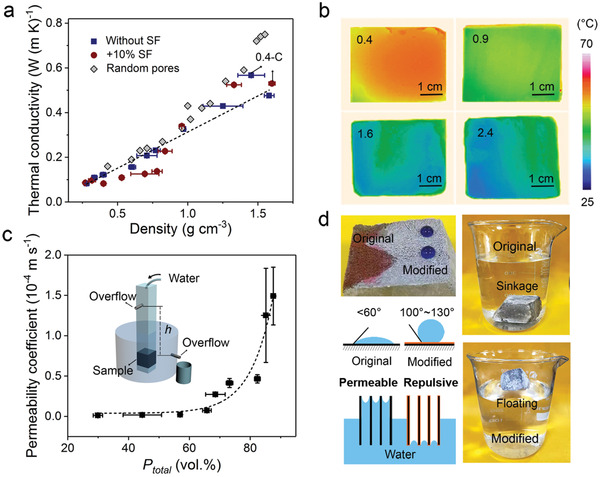
Multifunctional characteristics of wood‐like cement. a) Variations in the thermal conductivity coefficient of wood‐like cement at the transverse profile as a function of nominal density. 0.4‐C indicates the cement made from slurries with a W/C of 0.4 but without ice‐templating treatment. The data for randomly open‐celled porous cement materials are also shown for comparison.^[^ ^35^, ^36^ ^]^ b) Infrared images of cement made from slurries with differing W/C values of 0.4, 0.9, 1.6, and 2.4 placed on a heating plate of 100 °C. c) Dependence of the water permeability coefficient along the vertical direction on the total porosity *P*_total_ in wood‐like cement. The setup used for water permeability measurement is illustrated in the inset. d) Images and schematic illustrations showing the water permeable and repulsive nature of the cement before and after waterproofing treatment, along with the capillary attraction and repulsion effects of internal surfaces owing to the hydrophilic and hydrophobic characteristics. The data in panels (a) and (c) are obtained from at least three measurements for each set of samples and presented in form of mean ± standard deviation. The general varying trends are indicated by dashed curves for clarity.

**Figure** **4**a presents the thermal conductivity coefficient, *λ*, of the ice‐templated wood‐like cement along the normal direction of its lamellae as a function of the nominal density. The data for randomly porous cement‐based materials made by preforming methods are also shown for comparison.^[^ ^3^, ^35^, ^36^ ^]^ The ice‐templated cement displays monotonically decreasing *λ* with decreasing density, i.e., increasing porosity, in a wide range from ≈0.5 to lower than 0.1 W (m K)^−1^. The reduced *λ* at equal densities compared to the case of random pores indicates a more effective role of the unidirectionally porous architectures in diminishing the thermal conductivity. The good thermal insulation properties of the ice‐templated cement can be clearly visualized by the infrared images shown in Figure 4b. The temperatures of the cement are seen to be significantly decreased, by up to tens of degrees Celsius, at its top surfaces through a conducting distance (i.e., the sample thickness) of 20 mm with respect to the bottoms which are placed on a heating plate of 100 °C. The thermal insulation efficiency can be regulated by adjusting the solid load in the initial cementitious slurries with rising W/C leading to increasingly lower temperatures of samples.

In contrast to the insulation characteristics at the transverse profile, the unidirectionally interlamellar open pores offer abundant conduits for an effective transport in the ice‐templated cement along the vertical direction, i.e., the growth direction of ice during the freezing process. As shown in Figure 4c, the water permeability coefficient *k*, measured using the customized setup illustrated in the inset,^[^ ^37^ ^]^ increases monotonically with increasing porosity from minimal to around 1.5 × 10^−4^ m s^−1^. A sudden rise in *k* occurs after ≈70 vol% in total porosity, which is supposed to correspond to the threshold transition point for percolation.^[^ ^9^, ^38^ ^]^ Additionally, the interaction of the wood‐like cement with water can be modulated by tuning the chemically hydrophilic or hydrophobic characteristics of its internal surfaces of unidirectionally open pores. As shown in Figure 4d, the cement in its original form can easily absorb water because of its hydrophilic attribute (the contact angle of cement with water is typically lower than 60°).^[^ ^39^ ^]^ This leads to the shrinkage of the cement when immersing in water even when it has a lower nominal density than water. By contrast, the water can be effectively prevented from penetrating the pores in the cement via waterproofing treatment using an organosilicon agent. The contact angle with water can be improved to a range of 100°–130° as a result of the formation of hydrophobic residues at the surfaces of lamellae.^[^ ^40^ ^]^ By this means, the cement can even float on the water in case of W/C exceeding 0.7, which results in a high porosity of over 50 vol%, despite the fact that the majority of pores are open. This is attributed to the capillary repulsion effect of these pores resulting from their small dimensions and the hydrophobic nature of internal surfaces.^[^ ^41^ ^]^ Such a switchable behavior from being permeable to repulsive to water may offer the ice‐templated cement promise for applications as either pervious or waterproof building materials.

## 3 Discussion

The ice‐templating technique offers a viable approach for creating unidirectional micropores in materials, and has been widely employed in various material systems, e.g., ceramics, polymers, metals, and their composites.^[^ ^23^, ^24^, ^33^, ^34^, ^42^, ^43^, ^44^ ^]^ Recent attempts have also been devoted in ice‐templating cement materials.^[^ ^45^, ^46^ ^]^ It is critical for preserving the ice‐templated architectures by avoiding the collapse or re‐melting of the frozen body during the removal of ice. This invariably necessitates a freeze‐drying treatment to sublimate the ice, which requires long time processing of evacuation combined with refrigeration.^[^ ^24^ ^]^ For example, it typically takes more than 3 days to achieve an adequate drying of laboratory‐scale samples of tens of millimeters in dimension.^[^ ^42^, ^43^, ^44^, ^45^, ^46^ ^]^ The considerable energy consumption and time cost, together with the strict size limit by the vacuum chamber for freeze drying, make the ice‐templating technique inapplicable for the mass production in engineering. Additionally, in general the freeze‐dried green bodies need to be strengthened by adding certain amounts of organic adhesives.^[^ ^23^, ^45^, ^46^ ^]^ These organics are often removed via extra operation, e.g., high‐temperature heat treatment, which further complicates the fabrication process. The above problems are solved here by utilizing the unique self‐hardening behavior of cement through its in situ hydration reactions with water from the melted ice during the thawing process. This enables the retention of the ice‐templated architectures in cement materials without freeze drying or adding organic adhesives, and is thereby more time‐ and cost effective for practical applications. Similar strategies are believed to be pertinent to other material systems where self‐hardening can be realized by either hydration or nonhydration reactions, e.g., air hardening or UV polymerization. Indeed, we have verified the effectiveness of the current simplified ice‐templating technique in developing unidirectionally porous gypsum and bone cement for building and biomedical applications, which will be reported in detail elsewhere.^[^ ^47^ ^]^

For conventional cement materials, a mix with too much water in initial cementitious slurries usually leads to a low strength and poor durability in severe environmental conditions because of the easy penetration of aggressive ions into cement. The removal of excess water also tends to cause large shrinkage and even internal cracks. However, the effects of water additions are markedly different for the fabrication of the current wood‐like cement. The porosity in the final cement can be readily controlled by regulating the water content in slurries, i.e., from around 30 to over 80 vol% when W/C ranges from 0.4 to 2.8. During the ice‐templating process, the cement powders in slurries are expelled into the spacing between growing ice crystals to form the long‐range‐ordered lamellar structure. This is accompanied by the volumetric expansion caused by the freezing of water and thereby exerts squeezing forces to the cement powders.^[^ ^21^, ^48^, ^49^ ^]^ By such means, a dense packing of cement minerals can be achieved within the lamellae of wood‐like cement, as shown in Figure S7 (Supporting Information). As such, the lamellae are supposed to display similar resistance as conventional cement with low W/C to the penetration of aggressive ions. Additionally, large shrinkage, collapse, or internal cracking can be avoided during the removal of excess water from the cement owing to the dense packing and in situ hardening behavior of lamellae by hydration reactions (obvious shrinkage and collapse were only observed when W/C exceeds 2.8).

On the other hand, adding SF generally leads to a denser structure in conventional cement materials,^[^ ^50^, ^51^ ^]^ thereby resulting in an improvement of strength. A similar effect can be detected in the wood‐like cement as manifested by the slight decrease of porosity within lamellae, which can be obtained from total porosity *P*_total_ and interlamellar porosity *P*_inter_ following the relationship (*P*_total_ − *P*_inter_)/(1 − *P*_inter_), as shown in Figure S8 (Supporting Information). However, the strengthening effect is much less evident especially when the total porosities are concerned (Figure 3e). This is mainly caused by the fact that the interlamellar porosity, which accounts for a major proportion in the total, is controlled by the water content in initial slurries, but is nearly independent on whether SF is added (Figure 2f). Additionally, the cement minerals within lamellae exhibit a dense packing even without SF additions owing to the squeezing forces during freezing (Figure S7, Supporting Information). Therefore, the differences in the effects of SF additions for the wood‐like cement as compared to conventional cement materials are tightly associated with the ice‐templating technique for its fabrication.

With respect to the mechanical properties, the unidirectional alignment of lamellae functions to maximize the stiffness and strength along required directions considering that porous cement materials are most commonly subject to uniaxial loading conditions in practice. In the following, the strength of the wood‐like cement is formulated to discern the effects of the three types of pores, specifically the unidirectionally interlamellar open pores along with the open and closed intralamellar pores. The linear elastic fracture mechanics has been broadly employed in interpreting the strength of porous cement materials.^[^ ^52^, ^53^ ^]^ This is rationalized by the fact that the cement can be regarded as an ideally brittle material without any plastic deformability. Our analysis is based on the consideration of an ideally fully dense solid of cement which is an isotropic and homogeneous continuum. Considering a pre‐existing internal crack with constant half‐length, *a*, the strength of cement, *σ*, can be correlated to its elastic modulus *E* and nominal fracture energy *γ*, i.e., the energy needed for per unit area of crack propagation, following the relationship^[^ ^52^, ^53^ ^]^

(2) $$\begin{array}{c} \sigma \propto \sqrt{E\gamma /a} \end{array}$$

Then, the dependence of strength on the porosity can be understood in terms of the effects of different types of pores on the elastic modulus and nominal fracture energy (the length of presumed crack can be eventually eliminated in the derivation).

The elastic modulus *E* is dependent on the porosity *P* in porous solids following a power‐law relationship in the form as

(3) $$\begin{array}{c} E/E_{s}\propto {\left(1-P\right)}^{c} \end{array}$$

where *E*_s_ is the elastic modulus of a fully dense solid.^[^ ^54^, ^55^ ^]^ The exponent *c* is equal to 2 and 1, respectively, in case of isotropically and unidirectionally open pores, and generally takes a value of 2–3 for randomly closed pores depending on the relative density *ρ*_r_. It has been revealed that *c* approximates 3 when *ρ*_r_ is lower than 0.3, and decreases toward 2 with increasing *ρ*_r_.^[^ ^54^, ^55^ ^]^

Next, we derive the elastic modulus of the cement by isolating the effects from different types of pores using an equivalent element approach.^[^ ^56^, ^57^ ^]^ The intralamellar region, which contains merely closed pores, is defined as element A, as illustrated in Figure 2e. The elastic modulus *E*_A_ can be described using *E*_s_ as

(4) $$\begin{array}{c} E_{A}/E_{s}\propto {\left[1-\left(P_{\mathrm{total}}-P_{\mathrm{open}}\right)/\left(1-P_{\mathrm{open}}\right)\right]}^{c} \end{array}$$

The porosity (in randomly closed form) in element A, as depicted by the item (*P*_total_ − *P*_open_)/(1 − *P*_open_), is found to be lower than 0.4 in the majority of ice‐templated cement. This corresponds to a high relative density of over 0.6, thereby yielding a value of ≈2 for the exponent *c*.

The elastic modulus of the entire lamellae, represented using element *L*, can be obtained by incorporating the open intralamellar pores as

(5) $$\begin{array}{c} E_{L}/E_{A}\propto {\left[1-\left(P_{\mathrm{open}}-P_{\mathrm{inter}}\right)/\left(1-P_{\mathrm{inter}}\right)\right]}^{2} \end{array}$$

with (*P*_open_ − *P*_inter_)/(1 − *P*_inter_) describing the porosity (in randomly open form) in element L by treating element A as a dense solid. Similarly, the elastic modulus of the wood‐like cement, *E*, can be obtained by taking the unidirectional interlamellar pores into account as

(6) $$\begin{array}{c} E/E_{L}\propto \left(1-P_{\mathrm{inter}}\right) \end{array}$$

by treating element L as a dense solid. The combination of Equations (4)–(6) gives the relationship as

(7) $$\begin{array}{c} E/E_{s}\propto {\left(1-P_{\mathrm{total}}\right)}^{2}{\left(1-P_{\mathrm{inter}}\right)}^{-1} \end{array}$$

by approximating *c* as 2.

Additionally, it has been shown that the nominal fracture energy *γ* generally displays a decreasing trend with the increase of total porosity in porous cement materials and other mineral foams in an exponential fashion as

(8) $$\begin{array}{c} \gamma \;=\gamma_{0}\;e^{-bP_{\mathrm{total}}} \end{array}$$

where *γ*_0_ is the energy at zero porosity.^[^ ^53^, ^58^ ^]^ Parameter *b* was found to vary among different material systems with an average value of ≈7.^[^ ^58^ ^]^ Therefore, the relationship between the strength and porosity can be obtained by combining Equations (2), (7), and (8) as

(9) $$\begin{array}{c} \sigma \propto {\left[{\left(1-P_{\mathrm{total}}\right)}^{2}{\left(1-P_{\mathrm{inter}}\right)}^{-1}e^{-bP_{\mathrm{total}}}\right]}^{1/2} \end{array}$$

As shown in Figure 3f, Equation (9) offers a satisfied description about the strength of the ice‐templated cement with a goodness‐of‐fit *R* ^2^ exceeding 0.99.

**Figure 5:**
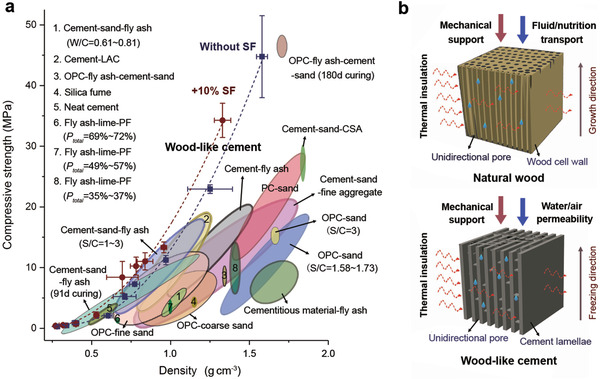
Comparison of wood‐like cement with natural wood and other porous cement materials.^[^ ^3^, ^4^, ^5^, ^6^, ^7^, ^8^, ^31^, ^43^, ^53^, ^59^, ^60^, ^61^ ^]^ a) Compressive strength and density for a wide range of porous cement‐based materials showing the relatively higher strengths of current wood‐like cement at equal densities. LAC: lightweight aggregate content; OPC: ordinary Portland cement; PF: polypropylene fiber; PC: Portland cement; CSA: concrete sludge aggregate; S/C: sand‐to‐cement ratio in weight. b) Schematic illustrations about the design strategies of natural wood and wood‐like cement in optimizing their mechanical and multifunctional properties associated with the unidirectionally porous architectures. The strength and density data of current wood‐like cement in panel (a) are presented in form of mean ± standard deviation.

**Figure** **5**a presents the strengths and densities of the wood‐like cement compared with other porous cement‐based materials reported in literature.^[^ ^3^, ^4^, ^5^, ^6^, ^7^, ^8^, ^31^, ^43^, ^53^, ^59^, ^60^, ^61^ ^]^ All the available data about porous cement, concrete, and other cement‐based materials fabricated by different methods are included for a comprehensive comparison. This is supposed to facilitate an explicit recognition about the properties and, particularly, the property advantages of current cement among various cement materials, thereby highlighting the effects of wood‐like architectures. It may also aid in the selection of cement materials in engineering for practical applications. It is seen that the wood‐like cement tends to exhibit higher strengths at equal densities than other cement materials. It even outperforms most of porous concrete materials which are strengthened with sands, aggregates, and other additives. Moreover, the strengths and densities of wood‐like cement can be regulated in wide ranges by tuning the water content in initial cementitious slurries for ice templating. In addition to the unidirectional alignment of architectures, the smaller sizes of pores, with tens of micrometers in thickness, are also beneficial for higher strengths considering that the pores in conventional porous cement‐based materials generally possess characteristic dimensions of sub‐ to several millimeters.^[^ ^4^, ^31^, ^61^ ^]^ On the other hand, the densities of the wood‐like cement may be decreased at fixed levels of strengths by the additions of SF despite its minimal influence on the microstructure and mechanical properties (Figures 2 and 3). This is attributed to the small density of SF, which is around 30% lower than that of cement (the densities of SF and cement are ≈2.21 and ≈3.15 g cm^−3^, respectively).^[^ ^9^ ^]^

The improved mechanical robustness of wood‐like cement is further accompanied by a unique multifunctionality with effective thermal insulation at the transverse profile and controllable water permeability along the vertical direction. Such configurations are virtually consistent with the requirements of properties for applications, e.g., as thermal insulation bricks or pervious pavements. These characteristics stem essentially from the implementation of the optimizing design principles of natural wood underlying its unidirectionally porous architectures.^[^ ^19^ ^]^ Figure 5b illustrates the similarities between the wood‐like cement with natural wood. Specifically, the thermal conduction between cement lamellae is principally carried out through their interconnections along the transverse direction. The alternative arrangement of lamellae and interlamellar pores can also lead to multiple reflection effect of thermal radiation for reduced heat transfer.^[^ ^62^ ^]^ This is reminiscent of the transversally thermal insulation properties in many species of wood.^[^ ^19^ ^]^ Additionally, the water permeability of wood‐like cement endowed by the well‐defined alignment and good continuity of open pores reproduces to a large extent the vertically conductive nature of wood. The cement can be readily adjusted from being water permeable to water repulsive by hydrophobic surface treatment. The capillary repulsion effect, which is associated with the small dimensions and the hydrophobic internal surfaces of pores,^[^ ^40^, ^41^ ^]^ can even lead to the floating of wood‐like cement on water. The outstanding combination of properties, including high strengths, low thermal conductivity, and controllable water permeability along specific directions, together with the feasible approach for easy production makes the wood‐like cement appealing for building applications, e.g., as thermal insulation bricks, masonry infills, and pervious pavements.

## 4 Conclusion

Wood‐like cement materials with unidirectionally porous architectures and varying porosities ranging from ≈30 to over 80 vol% have been fabricated based on a simplified ice‐templating technique. The ice‐templated architectures can be retained without freeze‐drying treatment by utilizing the self‐hardening behavior of cement through its in situ hydration reactions with water during the thawing process. The wood‐like cement contains different types of pores, specifically interlamellar pores and smaller intralamellar pores in either open or closed forms, and abundant interconnections bridging their lamellae. The strengths of the cement display a monotonic decreasing trend as the porosity increases. Such variations can be interpreted based on the linear elastic fracture mechanics by taking the effects of all types of pores into account according to an equivalent element approach. The wood‐like cement is additionally featured by lower thermal conductivities at the transverse profile as compared to randomly open‐celled porous counterparts together with good water permeability along the vertical direction. These properties can be regulated in wide ranges by controlling the porosities, which are negatively related to the solid loads in cementitious slurries. Moreover, the cement can be switched to be water repulsive by hydrophobic treatment as a result of the capillary repulsion effect of pores. The outstanding combination of higher strengths at equal densities than conventional porous cement‐based materials with the unique multifunctionality endows the wood‐like cement with a potential for a range of applications, particularly as new building materials. The feasible fabrication strategy, specifically in light of architectural retention based on the self‐hardening nature of ingredients, could significantly improve the time‐ and cost effectiveness of the ice‐templating technique and can also be applicable to other material systems.

## 5 Experimental Section

##### Preparation of Cementitious Slurries

Cementitious slurries with a wide range of water‐to‐cement ratios in weights (W/C) of 0.4, 0.7, 0.9, 1.1, 1.3, 1.6, 2.0, 2.4, and 2.8 were prepared by blending commercially available ordinary Portland cement powders (grade PO42.5 with a specific surface area of 342 m^2^ kg^−1^, United Cement Co., China) with deionized water using a rotary mixer. The viscosities of slurries were adjusted by adding 1 wt% hydroxypropyl methylcellulose powders (Meryer Co., China) with respect to cement. Such a type of admixture is frequently used in cement materials for reducing gravitational sedimentation in slurries, improving the water retention of cement, and enhancing the adhesion between cement powders after consolidation.^[^ ^27^ ^]^ A group of slurries were additionally mixed with SF powder (a specific surface area of ≈2 × 10^4^ m^2^ kg^−1^, Zhanpeng Co., China), which is a common additive in cement for promoting consolidation,^[^ ^4^, ^61^ ^]^ accounting for 10 wt% in the total of solid. The premixed slurries were then ball‐milled for 24 h for homogenization before use.

##### Fabrication of Wood‐Like Cement

The cementitious slurries were cast into square plastic molds with internal dimensions of 55 mm × 55 mm × 80 mm attached onto a copper plate. The bottom of the plate was connected with a copper rod, which was cooled by immersing into liquid nitrogen—a commonly used cooling agent in building engineering.^[^ ^27^ ^]^ Polydimethylsiloxane wedges with a slope angle of 25° were placed at the bottoms within the molds. This functions to create a horizontal temperature gradient in the slurries, in addition to the vertical one, from the thinner end to the thicker end of the wedge,^[^ ^22^ ^]^ as illustrated in Figure S1 (Supporting Information). After frozen, the specimens were allowed to slowly thaw by storing in a refrigerator set at a fixed temperature of 2 °C for 2 days. The ice‐templated architectures can be retained during the thawing process because of the in situ hardening of cement by the hydration reactions with the water formed from melted ice.^[^ ^27^ ^]^ Then the cement was immersed in water for 28 days at room temperature for achieving adequate curing. It was proved that hydration treatment for even longer time can hardly lead to additional hardening of the cement, as shown in Figure S2 (Supporting Information). The hardened samples were dried at 60 °C in an oven for at least 3 days until reaching a constant weight before characterization. For comparison, samples were also prepared from slurries with a W/C of 0.4 following the above procedures but without freezing treatment (0.4‐C).

##### Microstructural Characterization

SEM imaging was performed on the dried samples using a Leo Supra‐35 field‐emission scanning electron microscope operating at an accelerating voltage of 20 kV. The samples were sputter‐coated with a film of gold before imaging for electrical conduction. The 3D architectures were examined using an Xradia Versa XRM‐500 3D XRT system operating at an accelerating voltage of 80 kV. The stem of a *B. schmidtii* birch wood was also characterized for comparison. The samples were rotated by 360° with respect to the normal axis of profile for the X‐ray source and detector. A total of 1600 slices of 2D projections were taken for each sample by imaging per 0.225° during rotation and then were inverted into 3D volume renderings based on the Fourier back‐projection algorithm. The XRT images were processed and analyzed using the software of Avizo Fire 7.1 (Visualization Sciences Group, France).

##### Quantification of Porosities

The open porosities in the cement were determined based on the Archimedes’ principle. Specifically, a dried sample with an original weight *w*_0_ was immersed in methanol, which had a small molecular dimension of ≈4.4 Å and thus could easily permeate the cement, for 24 h to ensure the full filling of the open pores with methanol. The infiltrated sample was hung in the methanol and weighted, giving a weight *w*_1_. Then the sample was taken out from methanol and weighted again immediately after the surface was dried, giving a weight *w*_2_. The open porosity, *P*_open_, can be obtained as follows

(10) $$\begin{array}{c} P_{\mathrm{open}}=\left(w_{2}-w_{0}\right)/\left(w_{2}-w_{1}\right)\; \end{array}$$

The interlamellar porosities in the cement, *P*_inter_, were determined by analyzing the SEM images using the commercial software of Image Pro‐plus (Meyer Instruments Inc., TX). It was noted that the interconnections involved within the interlamellar pores were also taken into account in measuring *P*_inter_, as it was difficult to fully discriminate them from the pores, which may lead to some extent of overestimation in the interlamellar porosities. Nevertheless, such treatment is reasonable in formulating the strength considering that the load is principally carried by the lamellae along the vertical direction in the cement.

For quantifying the total porosities, *P*_total_, the true density of hardened cement by eliminating the involved pores, *ρ*_true_, was measured based on the Le Chatelier flask method, in accordance with ASTM Standard C188‐15.^[^ ^63^ ^]^ This was accomplished by manually grinding the cement into fine powders with an agate mortar. The powders were filtered using a sieve with a mesh size of 0.9 mm and dried at 110 °C for 1 h before use. A specific gravity bottle was infilled with anhydrous kerosene of around 1 mL and placed in a thermostat water bath of 20 °C for 30 min. The original volume of anhydrous kerosene was recorded as *V*_1_. Cement powders with the mass *m* were slowly added into the bottle with the air inclusions in powders removed by shaking the bottle during addition. The bottle was placed in the water bath for another 30 min and then measured, giving the volume *V*_2._ The true density of cement could thus be obtained as *ρ*_true_ = *m*/(*V*_2_ − *V*_1_). Accordingly, the total porosity can be calculated following the relationship

(11) $$\begin{array}{c} P_{\mathrm{total}}=\;1-\rho_{\mathrm{nominal}}/\rho_{\mathrm{true}} \end{array}$$

where *ρ*_nominal_ is the nominal density of cement determined by normalizing its weight to volume.

##### Mechanical Testing

Uniaxial compression tests were performed on rectangular samples of 5 mm × 5 mm × 10 mm in dimension with a fixed strain rate of 10^−3^ s^−1^ at room temperature. These samples were extracted from the dried cement using a STX‐202A precision diamond wire saw (Kejing Auto‐Instrument Co., China) with a low cutting speed of 0.1 mm min^−1^ to minimize damages introduced by the cutting process. The height direction of samples conformed to the freezing direction of slurries during ice templating. It was noted that generally cubic samples are used for determining the compressive mechanical properties of cement materials.^[^ ^64^ ^]^ However, the lamellae are easy to separate from each other during the cutting process of samples for mechanical testing in the current wood‐like cement, especially for that with large W/C, where the density of bridges is low. This can be effectively avoided by employing a larger height of samples with an aspect ratio of 2:1, conforming to the Chinese Standards for compression tests of ordinary cement materials GB/T 50081–2002.^[^ ^65^ ^]^ Indeed, the differences were tested to be statistically insignificant between the strengths of samples with different heights, as shown in Table S1 (Supporting Information). Samples made from relatively thick cementitious slurries with a W/C of up to 0.9 were tested using an Instron 5982 testing system equipped with an Instron 2580 load cell of 100 kN. An Instron E1000 testing system with a smaller Instron 2527‐302 load cell of 1 kN was used for samples with larger porosities, i.e., made from slurries with W/C exceeding 0.9.

##### Thermal Insulation Characterization

The thermal conductivities of wood‐like cement along the normal direction of its lamellae, i.e., perpendicular to the growth direction of ice during freezing, were measured according to the transient hot‐wire method using a TC‐3000E thermal conductivity meter (Xiatech Co., China). The thermal insulation properties of samples were further visualized by infrared imaging using a TH9100WR thermo tracer (NEC Avio Infrared Technologies Co., Japan) at a detection distance of 1 m after placing them on a MTI‐250 heating plate (MTI Co., China) set at a constant temperature of 100 °C. All the samples had a constant thickness of 20 mm and were heated for a fixed time of 20 min before observation.

##### Water Permeability Measurement

The water permeability coefficient was measured in general accordance with the standard EN 12697‐19 using a customized setup shown in the inset in Figure 4c.^[^ ^37^ ^]^ A sample with cross‐sectional area *S* and thickness *L* was inserted into the bottom of a square tube and sealed with paraffin to avoid water leak at its periphery. The height direction of the sample was consistent with the freezing direction during ice templating. The tube was fixed onto an overflow tank and filled with water from its top entry at room temperature. The water flow was adjusted to achieve a constant surface height in the tube such that the water inlet would be equal to the outlet owing to the stable water permeability of sample. The total volume of water outlet, *Q*, was then measured using a graduated cylinder at such flow for a constant time, *t*, of 15 min. The water permeability coefficient, *k*, can be obtained following the relationship

(12) $$\begin{array}{c} k\;=QL/\left(SHt\right) \end{array}$$

where *H* is the water drop between the square tube and overflow tank. The waterproofing treatment of porous cement was accomplished by immersing an organosilicon agent containing 33.3 wt% sodium methylsiliconate in water (Kolosun Co., China) for 1 day. The samples were then dried at 60 °C for at least 2 days before characterization.

##### Statistical Analysis

At least three measurements were conducted for quantifying each type of porosities in the cement for each set of samples. Specifically, the measured area exceeded 1 mm^2^ for each sample in the SEM image analysis. For mechanical testing, at least three tests were conducted for each set of cement samples made from slurries with different W/C. The differences between the strengths of samples with different heights, i.e., 10 and 5 mm, were accessed using the two‐sided Student's *t*‐test at a 5% level of significance. For the characterization of thermal insulation and water permeability properties, at least three measurements were performed for each set of samples. All the measured data were evaluated and presented in form of mean ± standard deviation.

## Conflict of Interest

The authors declare no conflict of interest.

## Author Contributions

Z.L. and Z.Z. designed the research; F.W., Y.D., and Y.Z. fabricated the cement; F.W. and D.J. characterized the microstructure and mechanical properties; F.W., Y.D., and Y.Z. measured the thermal insulation and water permeability properties; all the authors analyzed and discussed the data; and F.W. and Z.L. wrote the paper.

## Supporting information

Supporting Information

## Data Availability

The data that support the findings of this study are available from the corresponding author, Z.L., at zengqianliu@imr.ac.cn, upon reasonable request.
