# Supplementary material for: Wood‐Inspired Cement with High Strength and Multifunctionality
Source: Adv Sci (Weinh). 2020 Dec 23;8(3):2000096. doi: 10.1002/advs.202000096 (PMC7856898; doi:10.1002/advs.202000096)
Supplement: Supplementary file 1 — Supporting Information [file ADVS-8-2000096-s001.pdf]

© 2020 Wiley-VCH GmbH

ADVANCED SCIENCE
Open Access

Supporting Information

for *Adv. Sci.,* DOI: 10.1002/advs.202000096

Wood-Inspired Cement with High Strength and Multi-Functionality

Faheng Wang, Yuanbo Du, Da Jiao, Jian Zhang, Yuan Zhang, Zengqian Liu & Zhefeng Zhang**

Supporting Information

Wood-Inspired Cement with High Strength and Multi-Functionality

Faheng Wang, Yuanbo Du, Da Jiao, Jian Zhang, Yuan Zhang, Zengqian Liu &*
*Zhefeng Zhang**

F. Wang, Dr. D. Jiao, J. Zhang, Y. Zhang, Prof. Z. Liu, Prof. Z. Zhang

Shi-Changxu Innovation Center for Advanced Materials, Institute of Metal Research, Chinese Academy of Sciences, Shenyang 110016, China

E-mail: zengqianliu@imr.ac.cn; zhfzhang@imr.ac.cn

F. Wang

Nano Science and Technology Institute, University of Science and Technology of China, Suzhou 215123, China

F. Wang

Jihua Laboratory, Foshan 528200, China

Y. Du

School of Transportation Science and Engineering, Harbin Institute of Technology, Harbin 150090, China

Prof. Z. Liu, Prof. Z. Zhang

School of Materials Science and Engineering, University of Science and Technology of China, Hefei 230026, China

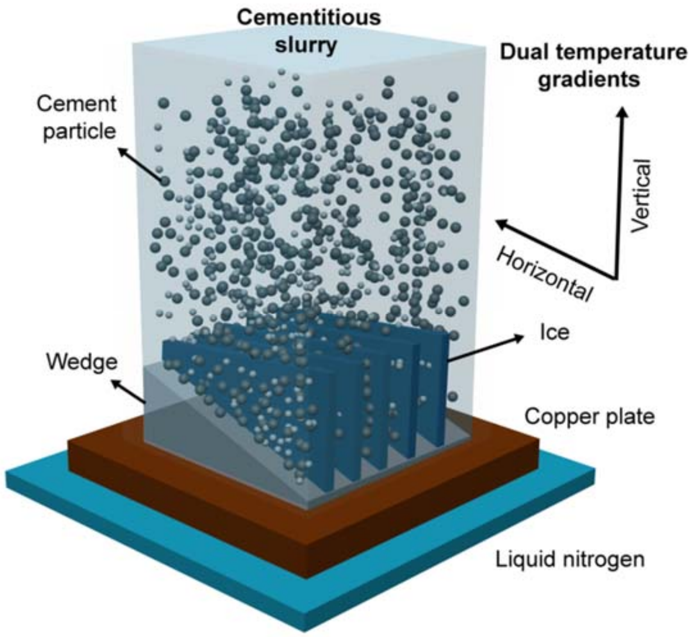

Figure S1. Schematic illustration about the ice-templating treatment of cementitious slurries for the fabrication of wood-like cement and the bidirectional growth of ice crystals during the freezing process. The arrows indicate the dual temperature gradients from the bottom to the top in the mold and from the thinner end to the thicker end of the wedge in the slurry.

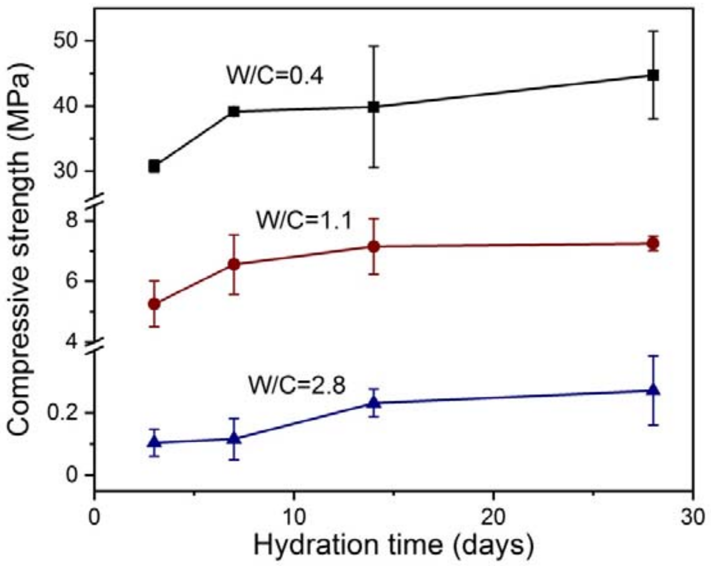

Figure S2. Representative variations in the compressive strengths for the wood-like cement produced from slurries with differing water-to-cement ratios (W/C) of 0.4, 1.1 and 2.8 as a function of the hydration time (for 3, 7, 14 and 28 days) during the curing process. The strengths demonstrate a continuously increasing trend with the hydration time and become relatively constant after 28 days. Hydration treatment for even longer time can hardly lead to obvious additional hardening in the cement. The compressive strengths were measured using rectangular samples of 5 mm × 5 mm × 10 mm in dimension with a fixed strain rate of 10^{-3} s^{-1} at room temperature. At least three tests were performed for each set of samples with the data presented in form of mean ± standard deviation.

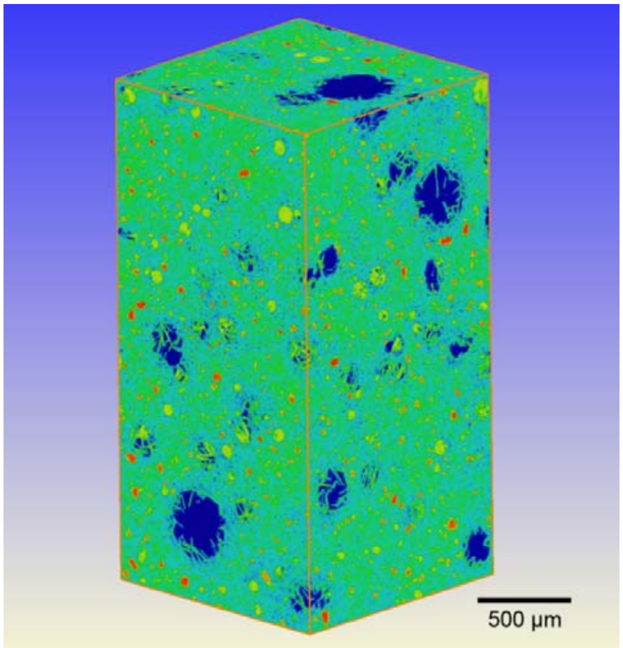

Figure S3. X-ray tomography volume renderings of the cement produced from cementitious slurry with W/C of 0.4 without ice-templating treatment (0.4-C). The 3-D micrograph clearly reveals the isometric geometries and random distribution of pores along with the existence of large spherical voids with diameters exceeding 300 μm in the cement. The pores in the cement are indicated with blue color.

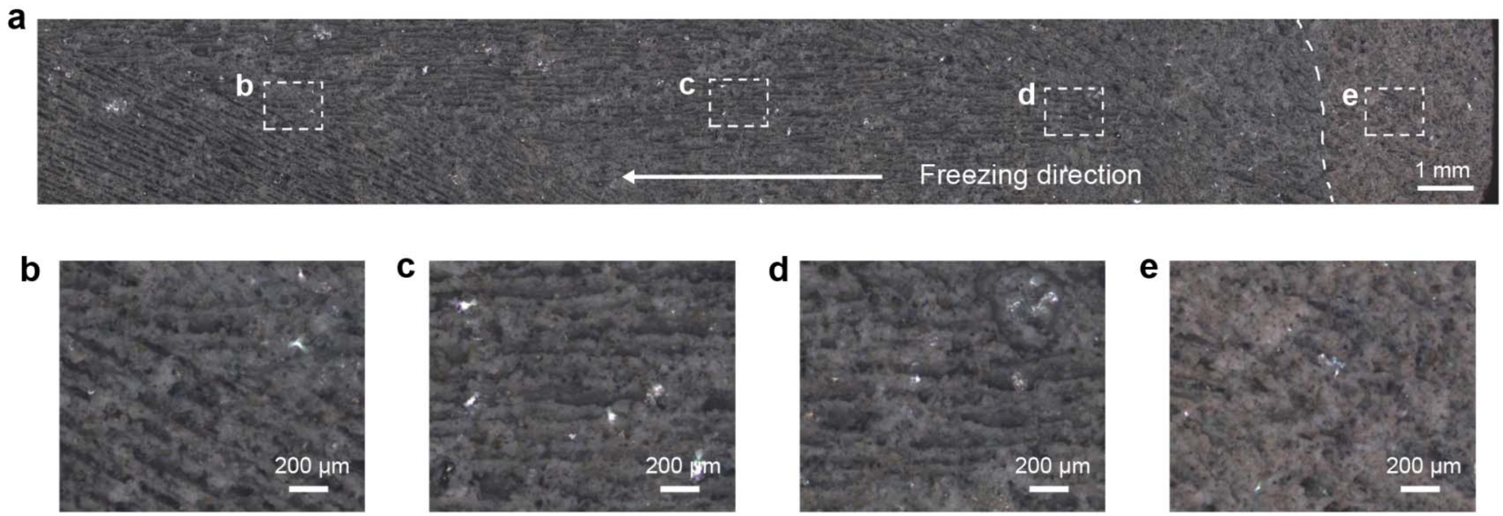

Figure S4. Representative cross-sectional optical images of the ice-templated cement produced from cementitious slurry with W/C of 1.3. (b-e) show the magnified morphologies of the local regions at differing distances away from the bottom end of sample as indicated by the dashed boxes in (a). The structural inhomogeneity is seen to mainly occur close to the bottom of sample, *i.e.*, near the end of wedge in the mold during the freezing process, and become markedly less evident or even indiscernible in the bulk of cement. The boundary of the bottom region with obvious inhomogeneity is roughly designated by the dashed curve.

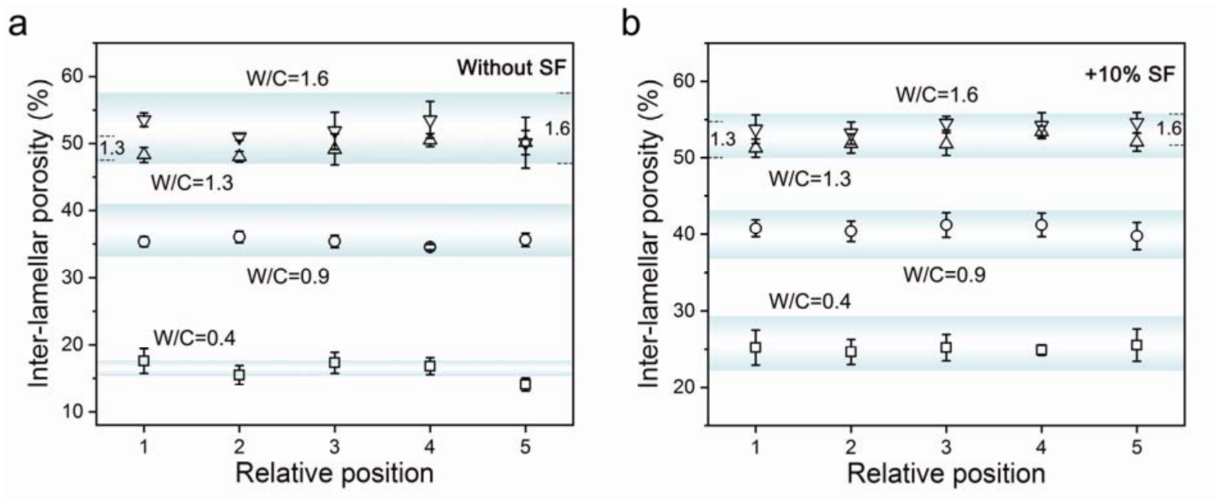

Figure S5. Variations in the inter-lamellar porosities of different sections at various positions for the ice-templated wood-like cement produced from slurries with W/C of 0.4, 0.9, 1.3 and 1.6. The numbers 1-5 denote the different sections with equal intervals between adjacent ones from the bottom to the top of samples along their height direction, *i.e.*, freezing direction during fabrication. The ranges (in form of mean ± standard deviation) of inter-lamellar porosities for the entire cement are indicated by the shaded regions. It is seen that the inter-lamellar porosities at almost all these sections locate well within the ranges for the entire cement with no obvious differences discernable between different positions for given W/C.

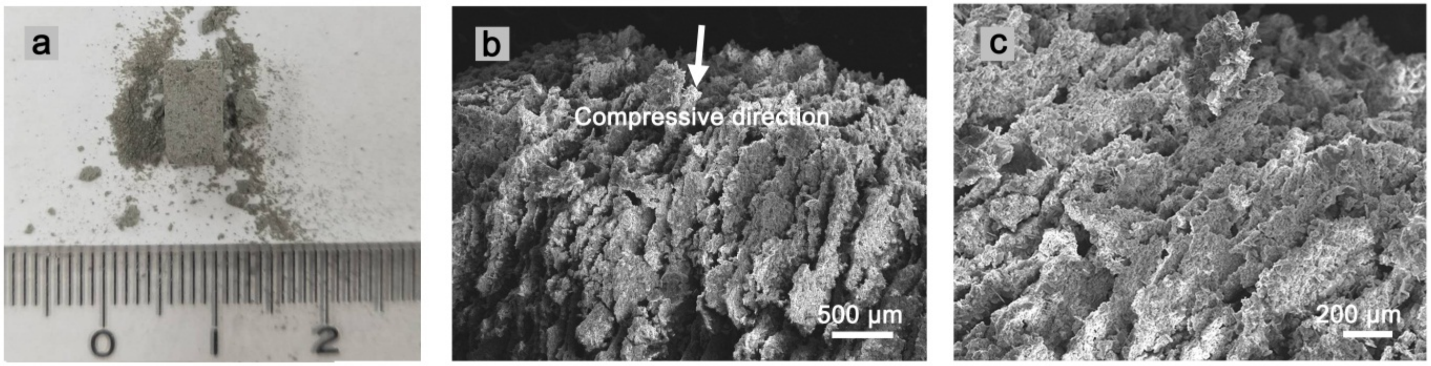

Figure S6. (a) Overall appearance and (b, c) SEM micrographs of a wood-like cement sample (made from slurries with W/C of 2.4) after compression test. The SEM micrographs were taken from the top area of sample contacting with the compressive platen with the magnification increased from (b) to (c). The large nominal plastic deformation of the sample originates essentially from the continuous brittle collapse of its lamellae.

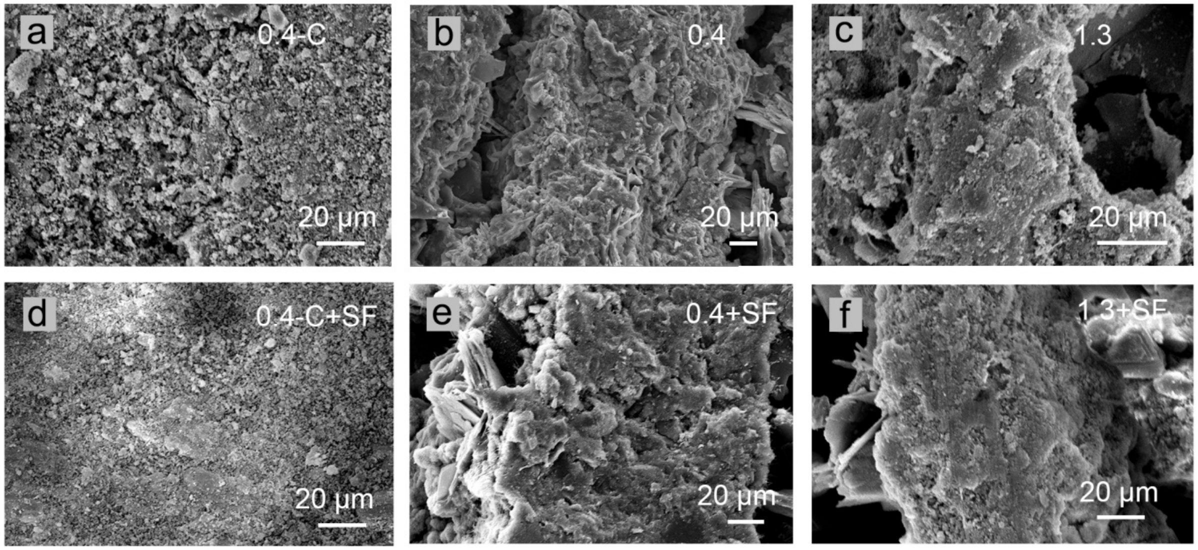

Figure S7. SEM micrographs of the wood-like cement (a-c) without and (d-f) with silicon fume (SF) additions made from slurries with W/C of 0.4 and 1.3. The cement minerals display a dense packing within the lamellae because of the squeezing forces exerted by ice crystals during the freezing process. The microstructures of unfrozen cement (0.4-C) are also presented for comparison.

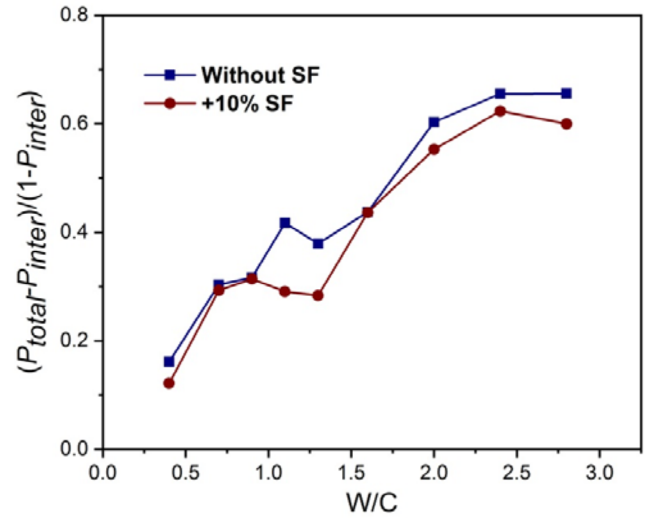

Figure S8. Variations in the average porosities within lamellae $(P_{total} - P_{inter})/(1 - P_{inter})$ in the wood-like cement with and without SF additions made from slurries with different W/C.

Table S1. Comparison of the strengths of wood-like cement measured using samples with different dimensions revealing statistically insignificant differences at a 5% level of significance using the two-sided Student's *t*-test. The water-to-cement ratios of initial slurries are 0.9, 1.1, 1.3 and 1.6. I: 5 mm × 5 mm × 5 mm; II: 5 mm × 5 mm × 10 mm.

Samples	Average strength (MPa)	Standard deviation (MPa)	Calculated *t*-value	*t*-table value	Testing result
0.9-I (n=3)	11.98	0.21	1.45344	2.7764	Insignificant
0.9-II (n=3)	10.53	1.32			
1.1-I (n=3)	7.35	0.91	0.48988	2.7764	Insignificant
1.1-II (n=3)	7.62	0.66			
1.3-I (n=5)	6.01	0.95	1.96982	2.4469	Insignificant
1.3-II (n=3)	4.87	0.67			
1.6-I (n=3)	3.06	1.13	1.27156	2.2622	Insignificant
1.6-II (n=8)	2.08	0.09			

Formation mechanisms of products during hydration reactions

The products of the hydration reactions of cement with water have been revealed to be principally composed of minerals of calcium hydroxide ($Ca(OH)_2$) and ettringite ($3CaO \cdot Al_2O_3 \cdot 3CaSO_4 \cdot 31H_2O$) which typically have hexagon and needle-like shapes, respectively, along with the calcium-silicate-hydrate gels ($3CaO \cdot 2SiO_2 \cdot 3H_2O$).[S1-S3] The detailed chemical reactions for the formation of these phases are as following:[S1-S3]

$$2(2CaO \cdot SiO_2) + 4H_2O \rightarrow 3CaO \cdot 2SiO_2 \cdot 3H_2O + Ca(OH)_2,$$

$$2(3CaO \cdot SiO_2) + 6H_2O \rightarrow 3CaO \cdot 2SiO_2 \cdot 3H_2O + 3Ca(OH)_2,$$

$$3CaO \cdot Al_2O_3 \cdot 6H_2O + 3(CaSO_4 \cdot 2H_2O) + 19H_2O \rightarrow 3CaO \cdot Al_2O_3 \cdot 3CaSO_4 \cdot 31H_2O.$$

References:

[S1] J. W. Bullard, H. M. Jennings, R. A. Livingston, A. Nonat, G. W. Scherer, J. S. Schweitzer, K. L. Scrivener, J. J. Thomas, *Cem. Concr. Res.* **2011**, *41*, 1208.

[S2] H. F. W. Taylor, *Cement Chemistry*, Thomas Telford, London, UK **1997**.

[S3] T. Matschei, B. Lothenbach, F. P. Glasser, *Cem. Concr. Res.* **2007**, *37*, 551.
